# Immunophenotypic p14 and p16 correlations with CDKN2A mutations in primary multiple and familial melanoma: An observational study

**DOI:** 10.1097/MD.0000000000036756

**Published:** 2023-12-22

**Authors:** Luana-Andreea Boşoteanu, Emma Gheorghe, Mariana Aşchie, Georgeta Camelia Cozaru, Mariana Deacu, Cristian Ionuț Orășanu, Mădălina Boşoteanu

**Affiliations:** a Department of Dermatovenerology, “Elias” Emergency University Hospital, Bucharest, Romania; b Institute of Doctoral Studies, Doctoral School of Medicine, “Ovidius” University of Constanţa, Constanţa, Romania; c Department of Dermatology, “Sf. Apostol Andrei” Emergency County Hospital, Constanţa, Romania; d Department of Histology, Faculty of Medicine, “Ovidius” University of Constanţa, Constanţa, Romania; e Clinical Service of Pathology, “Sf. Apostol Andrei” Emergency County Hospital, Constanţa, Romania; f Department of Pathology, Faculty of Medicine, “Ovidius” University of Constanţa, Constanţa, Romania; g Department VIII – Medical Sciences, Academy of Romanian Scientists, Bucharest, Romania; h Center for Research and Development of the Morphological and Genetic Studies of Malignant Pathology (CEDMOG), Constanța, Romania.

**Keywords:** CDKN2A mutation, familial melanoma, FISH, immunohistochemistry, multiple primary melanoma

## Abstract

Melanoma represents an aggressive malignant tumor, encapsulating frequent loss of differentiation markers, with familial melanoma constituting a relatively commonly encountered entity, in direct relationship with cyclin-dependent kinase inhibitor 2A (CDKN2A). The present study aims to identify the association between the immunohistochemical p14–p16 profile, the molecular CDKN2A findings and clinically diagnosed familial or multiple primary melanomas (MPM). We conducted a 5-year retrospective cross-sectional study, on patients diagnosed with familial or MPM. P14 and p16 immunohistochemical staining has been applied on the selected surgical specimens simultaneously with the performance of fluorescence in situ hybridization (FISH) CDKN2A testing. 13 out of the 23 included cases displayed p14 and/or p16 immunohistochemical absence and the main positive relationships were encountered between CDKN2A homozygous deletion and p14 ± p16 negative immunoreactions. Cases with exclusive p16 absent reaction (n = 7) were more frequently associated with the presence of distant metastases (85.71%), while samples with exclusive p14 immunohistochemical loss exhibited more favorable histopathological prognostic markers. The average percentage of p16-stained nuclei in the superficial dermis and the deep dermis were equal (29.54% for each), therefore infirming its potential predictive and/or prognostic utility. The present study is the first of its type to approach the clinical, evolutionary and immunophenotypic correlations between p14–p16 immunohistochemical testing, CDKN2A molecular biology pattern, familial melanoma and spontaneous MPM in a cohort of Romanian patients. This analysis highlighted the value of singular p16 immunohistochemical absence as a predictor for aggressive biological behavior and unfavorable prognosis in familial melanoma and/or MPM, in comparison with the exclusive loss of p14, indifferent to the histopathological subtype. The present study emphasizes the utility of immunohistochemistry as a less expensive method of complementing the current testing arsenal and could represent the starting point for the elaboration of tailored diagnostic and therapeutic algorithms, based on the discovered p14–p16-CDKN2A significant correlation.

## 1. Introduction

Melanoma represents an aggressive malignant tumor, encapsulating frequent loss of differentiation markers, associated with accelerated evolution, and limited survival rates. The available data regarding its incidence highlight a 2-fold increase every decade.^[1]^ Familial melanoma constitutes a relatively commonly encountered entity in the global nosological context of melanoma, being situated in direct relationship with cyclin-dependent kinase inhibitor 2A (CDKN2A) and CDK4, 2 genes essentially involved in cellular division, whose potential alterations raise the risk of occurrence of melanocytic malignancies.^[2]^

CDKN2A mutation is generally correlated with young age at diagnosis, familial aggregation and cases of multiple primary melanoma (MPM).^[3]^ Moreover, it encodes 2 tumor suppressive proteins—p14 and p16—and, up to this date, the documented immunohistochemical expression of these markers has been significantly associated with familial cases of mucous and cutaneous melanoma.^[4]^

As it is known, the CDKN2A gene, often referred to as p16 or INK4a/adenosine diphosphate-ribosylation factor (ARF), is located in the chromosomal region 9p21. CDKN2A, a tumor suppressor gene, is inactivated by homozygous deletions with high frequency in a variety of human primary tumors. Loss of the CDKN2A gene results in cellular proliferation and dysregulation of pro-apoptotic pathways.^[5]^ Those individuals that carry INK4a/ARF germline mutations and, therefore, whose p16^INK4a^ structure is functionally altered, are supplementarily affected by modifications in p14^ARF^. Mutations affecting both tumor suppressor proteins (p14 and p16) generate cellular clones that are capable of surviving consequent deoxyribonucleic acid damage, thus accumulating deoxyribonucleic acid injuries responsible for tumor progression and expression.^[6]^

The importance of the topic also resides in the oncological syndrome present in CDKN2A mutation carriers, that link melanoma and pancreatic cancer; thus, a CDKN2A-positive individual that has already developed melanoma has a 38-fold increased risk of associating pancreatic malignancies.^[7]^

Information related to the association between the immunohistochemical p14–p16 profile, the family history of melanoma and multiple primary melanomas is not fully elucidated, therefore the aim of the present study is to identify the role of immunohistochemical in accurately establishing the diagnosis and follow-up approach in the aforementioned categories. Furthermore, the objectives include the correlation between the CDKN2A tumoral status and clinic-pathological melanoma characteristics (such as anatomical site, histopathological subtype, Breslow index, mitotic rate, etc.) and the possibility of using CDKN2A variations as potential prognostic markers in melanoma.

## 2. Methods

We conducted a retrospective cross-sectional study, over a period of 5 years (2018–2022), on patients diagnosed with mucous and/or cutaneous melanoma, with a positive family history of melanoma, or personal history of primary malignant melanocytic lesions. Thus, we scanned the physical records within the archive and the digital database of the Clinical Service of Pathology, Emergency Clinical County Hospital of Constanţa, located in South-Eastern Romania.

The inclusion criteria comprised adult patients (over the age of 18 at diagnosis), with mucous and/or cutaneous melanoma confirmed histopathologically in the context of familial melanoma and/or multiple primary melanomas. The exclusion criteria referred to underage patients, with melanoma detected in visceral sites, neither with other affected family members, nor with previously diagnosed primary melanomas.

Afterward, epidemiological and clinical data, such as sex, age at diagnosis, area of residence, anatomical site of melanoma development, number of pigmentary lesions and elected treatment were extracted from the medical files. This information was evaluated by the attending dermatologist and the required excisions were performed by the plastic surgeon.

The tissue samples obtained after the elliptical surgical excision and re-excision of the primary tumor were described from a macroscopical point of view and processed according to standard protocols, to acquire hematoxylin and eosin stained slides. The latter were evaluated by experienced pathologists within the Clinical Service of Pathology and essential characteristics (histopathological melanoma subtype, Breslow index, mitotic rate, presence/absence of lymphovascular and perineural invasion, type of lymphocytic infiltrate) were noted. Moreover, the v-raf murine sarcoma viral oncogene homolog B1 (BRAF) status of the selected cases was investigated in an external laboratory and the results were labeled positive/negative, according to the specific genetic findings.

Supplementarily, the immunohistochemical technique for p14 and p16 was performed in line with the protocol provided by the manufacturer (Table 1), using 4 µm-thick sections derived from formalin-fixed paraffin-embedded specimens. For p14, the nuclear reaction was assessed in at least 100 nuclei comprised in the tumoral proliferation, while p16 was considered positive when displaying intense nuclear and cytoplasmic reactions. Aiming to maintain the accuracy of the method and further results, positive control slides were provided for every determination.

**Table 1:** Immunohistochemical and molecular biology panel.

Immunohistochemical antibody	Clone	Manufacturer	Dilution	Host, clonality
p16-INK4	MX007	Master Diagnostica	RTU (ready-to-use) 7 mL	Mouse, monoclonal
p14ARF	NB200-111	NovusBio	1:250	Rabbit, polyclonal
FISH probe	Clone	Manufacturer	Dilution	Additional materials
SPEC CDKN2A/CEN 9 Dual Color Probe	ZytoLight®	ZytoVision	RTU (ready-to-use) 0.2 mL	ZytoLight FISH Implementation Kit

Abnormal results regarding p16 protein immunotests comprised the absence of staining in both the nuclear and the cytoplasmic component, as well as exclusive cytoplasmic staining, slides with weak staining intensity and patterns of focal or patchy staining.^[8]^

Molecular biology tests investigating the CDKN2A status were executed at the Center for Research and Development of the Morphological and Genetic Studies of Malignant Pathology. Dual-color fluorescence in situ hybridization (FISH) for CDKN2A and chromosome 9 centromere (Table 1) was performed on 23 cases to establish percentage values for p16/CDKN2A deletion on formalin-fixed paraffin-embedded material.

Slides were examined and images were obtained using an epifluorescent microscope (Zeiss microscope—Axio Imager.M2, Zeiss) equipped with appropriate filters and an image-analysis system (MetaSystem Isis, Altlussheim). The SPEC CDKN2A/CEN 9 Dual Color Probe is a mixture of an orange fluorochrome direct labeled CEN 9 probe specific for the classical satellite III region of chromosome 9 (D9Z3) at 9q12 and a green fluorochrome direct labeled SPEC CDKN2A probe, specific for the CDKN2A gene at 9p21.3. A normal interphase nucleus is distinguished by the presence of 2 orange and 2 green signals.^[9]^ We evaluated at least 100 non-overlapped intact interphase nuclei, characterized by uniform 4′,6-diamidino-2-phenylindole staining with intact nuclear contours, of consecutive cells in at least 2 different areas of the section for each sample. Contrastively, a cell with deletion of the CDKN2A gene locus displays a reduced number of green signals.^[9]^ Specimens containing ≥15 nuclei that lacked both signals for CDKN2A (no green signal) and contained at least 1 signal for chromosome 9 centromere (at least 1 orange signal) were considered positive for homozygous deletion. Eventually, partial deletions of the CDKN2A gene could be expressed as a combination of the normal signal pattern and green signals of reduced size.^[9]^

The entire informational panel derived from the present research was summarized and standardized in a Microsoft Excel sheet (Microsoft, WA). The same program was also employed to process the data and to calculate the *P*-value for the appropriate associations; a *P*-value inferior to .05 was considered statistically significant.

Confidentiality of the patients was respected during the deployment of the present research, in compliance with the regulations stated by the Declaration of Helsinki and the study was approved by the Ethics Committee of the “Ovidius” University of Constanta, Romania, for studies involving humans. Each person signed a form granting their informed and assumed consent prior to each hospitalization at our institution.

## 3. Results

**Figure 1.**
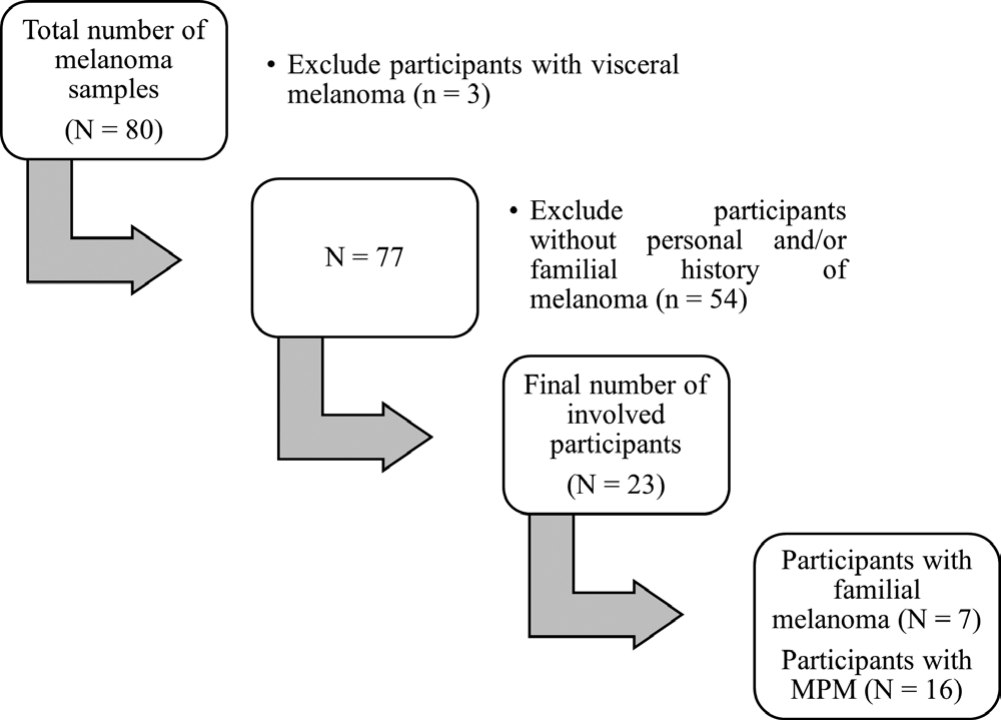
Flowchart of data selection.

After the preliminary search among medical records, we identified 80 patients with a histopathological diagnosis of melanoma. After the application of inclusion criteria, we excluded 3 patients with visceral melanoma and 54 others who did not exhibit either personal, or familial characteristics superposable to CDKN2A positivity. Hence the final group over the period of 5 years comprised 23 subjects—7 individuals emerging from families with at least one other first-degree relative diagnosed with melanoma, and 16 patients with MPM (Fig. 1).

### 3.1. Demographic, clinical, histopathological, and genetic characteristics

The mean age at diagnosis was 62.3 ± 14.7 years old, with an almost equal gender distribution (male:female ratio 1.09:1). A prevalence of cases was encountered in the 8th (30.43%, n = 7) and 7th (21.74%, n = 5) decade of life, and more than half of the patients resided in urban areas (60.87%, n = 14). The most common anatomic site was the anterior/posterior thorax (39.13%, n = 9), followed closely by the involvement of the head and neck cutaneous areas (34.78%, n = 8). The demographic and clinical particularities of the studied patients are depicted in Table 2.

**Table 2:** Demographic and clinical characteristics of the included participants (n = 23).

*Gender*	
Male	47.82% (n = 11)
Female	52.17% (n = 12)
*Age*	
* Mean age:*	*62.3 ± 14.7*
31–40	13.04% (n = 3)
41–50	17.39% (n = 4)
51–60	8.69% (n = 2)
61–70	21.73% (n = 5)
71–80	30.43% (n = 7)
81–90	8.69% (n = 2)
*Area of residence*	
Urban	60.86% (n = 14)
Rural	39.13% (n = 9)
*Anatomic site of primary melanoma*	
Head & neck	34.78% (n = 8)
Upper extremities	21.73% (n = 5)
Lower extremities	4.34% (n = 1)
Anterior/posterior thorax	39.13% (n = 9)

Regarding the histopathological aspects of the selected melanoma fragments (Table 3), the majority of cases corresponded to superficial spreading melanomas (56.52%, n = 13), while nodular melanomas were diagnosed in 9 cases (39.13%). The mean Breslow depth measured 4.88 ± 3.92 mm, a value derived from the preponderance of tumoral populations surpassing 4.01 mm on vertical sections (47.82%, n = 11). Ulceration was predominant in the analyzed cases and equally frequent as lymphovascular invasion (56.52%, n = 13), while perineural invasion and regression were only encountered in 21.73% (n = 5) and 17.39% (n = 4) of cases, respectively. The average mitotic rate determined in the selected melanoma samples was 3.04 ± 3.55 per mm^2^, most of the cases harboring between 1.00 to 1.99 mitoses/mm^2^ (34.78%, n = 8) and ≥5.00 mitoses/mm^2^ (34.78%, n = 8).

**Table 3:** Histopathological characterization of the evaluated melanoma lesions (n = 23).

*Histopathological melanoma subtype*	
Superficial spreading melanoma (SSM)	56.52% (n = 13)
Nodular melanoma	39.13% (n = 9)
Acral lentiginous melanoma	4.34% (n = 1)
*Breslow depth (mm*)	
* Mean Breslow depth*	*4.88 ± 3.92*
* *≤1.00	21.73% (n = 5)
* *1.01–2.00	13.04% (n = 3)
* *2.01–4.00	17.39% (n = 4)
* *≥4.01	47.82% (n = 11)
*Ulceration*	
Present	56.52% (n = 13)
Absent	43.47% (n = 10)
*Mitotic rate (per mm^2^*)	
* Mean mitotic rate*	*3.04 ± 3.55*
0	17.39% (n = 4)
1.00–1.99	34.78% (n = 8)
2.00–4.99	13.04% (n = 3)
≥5.00	34.78% (n = 8)
*Lymphovascular invasion*	
Present	56.52% (n = 13)
Absent	43.47% (n = 10)
*Perineural invasion*	
Present	21.73% (n = 5)
Absent	78.26% (n = 18)
*Regression*	
Present	17.39% (n = 4)
Absent	82.61% (n = 19)

Moreover, BRAF genetic testing identified the prevalence of negative cases (60.86%, n = 14), associated with CDKN2A monosomy (21.42%, n = 3), disomy (28.57%, n = 4), homozygous (28.57%, n = 4) or heterozygous deletion (21.42%, n = 3). BRAF-wild type samples were majorly encompassed in the clinical global context of absent distant metastases at the time of diagnosis (78.57%, n = 11), with only a small percentage of cases corresponding to metastatic melanomas (21.42%, n = 3). In the latter subgroup, secondary tumoral determinations were diagnosed at the pulmonary, cerebral, hepatic and/or peritoneal levels. On the other hand, we identified 9 out of 23 cases that harbored BRAF gene mutations corroborated with CDKN2A homozygous deletion (33.33%), CDKN2A monosomic (33.33%) or disomic (33.33%) alterations. Among the BRAF-mutated specimens, 66.66% (n = 6) were associated with distant metastases located in the liver, brain, lung, peritoneum, and/or cutaneous organ.

### 3.2. Immunohistochemical findings on p14 and p16 proteins

The staining pattern of p14/p16-processed specimens was evaluated based on the positive or negative immunoexpression encountered at the cytoplasmic and/or nuclear levels and its intensity was noted according to the following scale: negative reaction (−, 0% positive cells), weak (+, 1–10% positive cells), moderate (++, 11–50% positive cells), and strong (+++, >50% positive cells).

**Figure 2.**
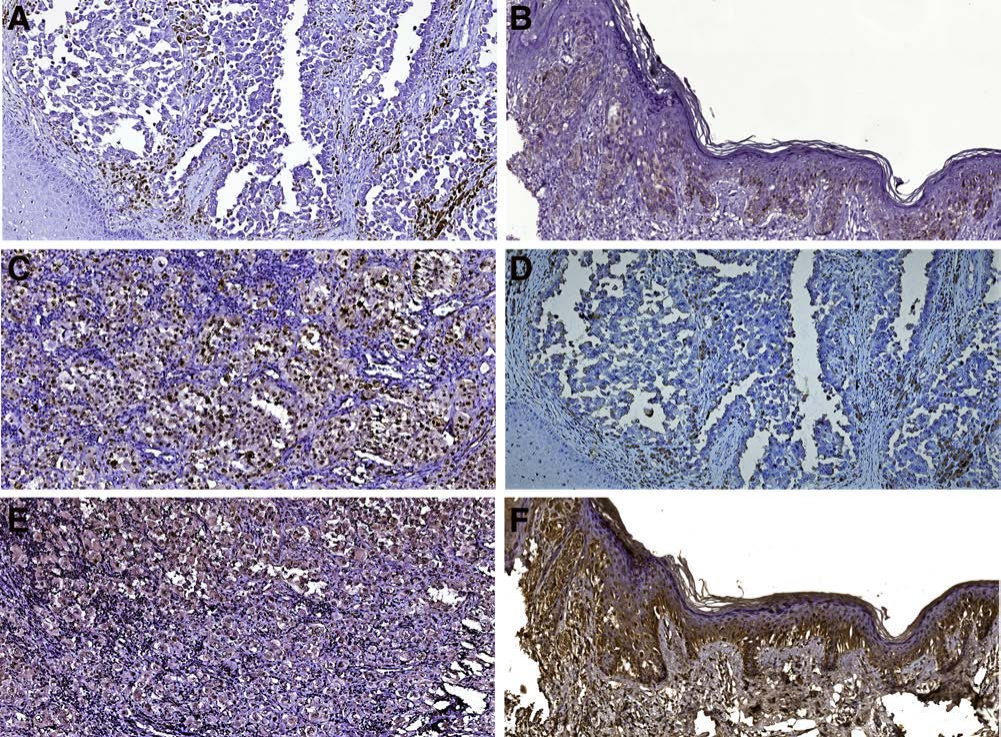
Immunohistochemical p14/p16 reactions encountered in the analyzed melanoma specimens. (A) Digital image of p16 absent reaction in a cutaneous melanoma specimen (−, ×10). (B) Digital image of cutaneous melanoma with moderate p16 expression in some nuclei (++, ×10). (C) Digital image of cutaneous melanoma showing strong p16 protein staining intensity (+++, ×10). (D) Digital image of cutaneous melanoma with loss of p14 expression (−, ×10). (E) Digital image of cutaneous melanoma revealing moderate intensity of the nuclear p14 staining (++, ×10). (F) Digital image of cutaneous melanoma with strong nuclear expression of p14 protein (+++, ×10).

Out of the 23 cases that met the eligibility criteria, 13 samples displayed p14 and/or p16 immunohistochemical absence. Cases with exclusive p16 absent reaction (n = 7, Fig. 2A) were more frequently associated with the presence of distant metastases (85.71%), as compared to 50% of the 4 specimens that only lost p14 expression (Fig. 2D).

The evaluation of p16 protein expression intensity displayed no case with weak staining, moderate intensity in 3 samples (Fig. 2B) and overexpression in 11 samples (Fig. 2C). On the other hand, the analysis of the p14 immunohistochemical reaction revealed weak intensity at the cytoplasmic level in 7 cases, moderate expression in 6 (Fig. 2E) and strong intensity in 4 tissue fragments (Fig. 2F).

The average Breslow value correlated with p16 loss and lack of p14 reaction was 6.79 mm and 5.37 mm, respectively.

The specimens that only displayed p16 absence encompassed superficial spreading melanomas (SSM, 42.85%), nodular melanomas (42.85%) and acral melanomas (14.28%), among which 71.42% were ulcerated. The group with p14 loss totaled 2 SSMs and 2 nodular melanomas, with an ulceration percentage of 25%. Those with loss of both protein markers included 2 SSMs.

Cases with exclusive p16 loss harbored an average mitotic rate of 5.28/mm^2^, compared to the average value of 2/mm^2^ recorded in the population with exclusive absent p14 immunoexpression.

Concerning the percentage of positive nuclei identified in p16-stained melanoma slides, their localization in the papillary dermis versus the reticular dermis did not harbor any statistically significant difference. The average percentage of stained nuclei in the superficial dermis was equal to the average percentage of positive nuclei in the deep dermis (29.54% for each), thus invalidating its potential prognostic and/or predictive utility.

### 3.3. Molecular findings on CDKN2A mutations through FISH technique (Fig. 3)

Heterozygous deletion of CDKN2A was encountered in 3 cases (13.04%, Fig. 4A), while homozygous deletions were detected in 7 specimens (30.43%, Fig. 4B). Disomy was also frequent, in 7 patients (30.43%, Fig. 4C) and CDKN2A monosomy was diagnosed via fluorescent in situ hybridization in 6 cases (26.08%, Fig. 4D).

**Figure 3.**
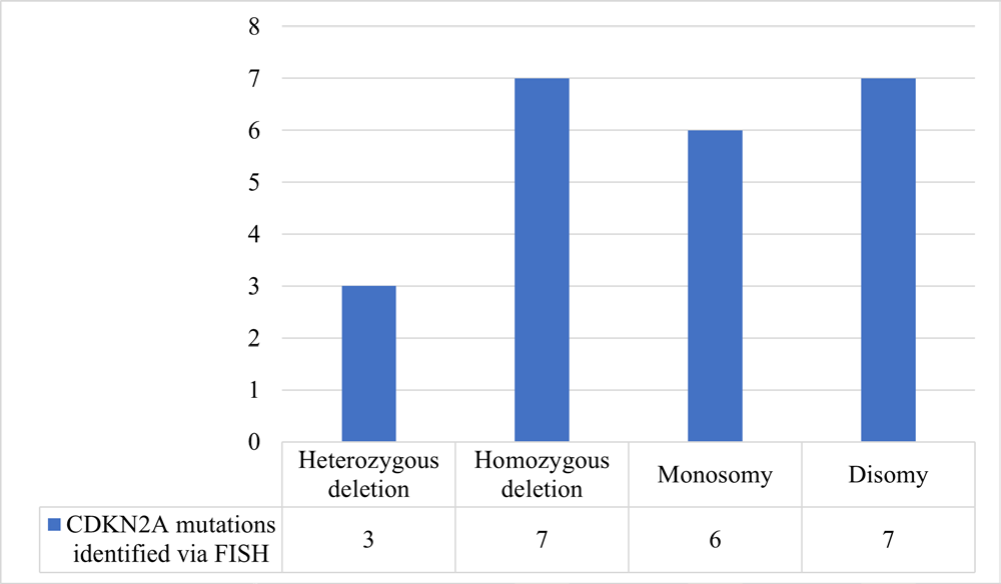
Distribution of CDKN2A mutations identified via FISH technique.

**Figure 4.**
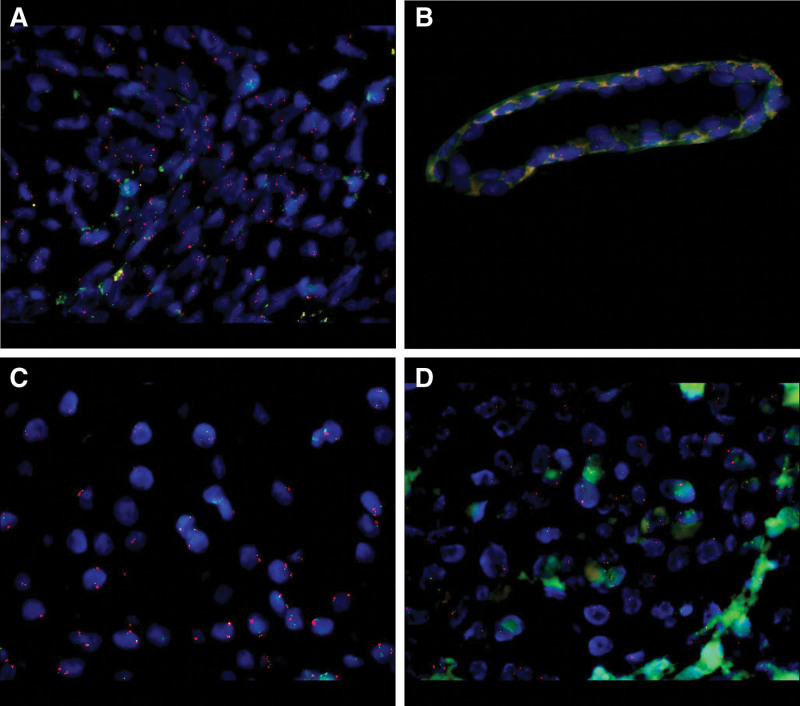
FISH technique applied to melanoma samples. (A) The image shows heterozygous CDKN2A deletion. (B) The picture displays homozygous CDKN2A deletion. (C) The image depicts CDKN2A disomy. (D) The image shows CDKN2A monosomy.

### 3.4. Association between the molecular CDKN2A status and immunohistochemical expression of p14 and p16

All 7 cases with CDKN2A homozygous deletion were associated with immunohistochemical loss of the p16 marker and 6 out of the 7 cases of CDKN2A homozygous deletion were also associated with p14 negative reactions. The other 2 cases that comprised the absence of p16 immunoexpression were correlated with CDKN2A monosomic mutations.

Among the samples with CDKN2A disomy, 5 cases presented moderate or strong immunohistochemical p14 and p16 stainings, while only 2 cases displayed negative p14 immunoexpression.

## 4. Discussion

To our knowledge, the present study is the first of its type to approach the clinical, evolutionary and immunophenotypic correlations between p14 and p16 immunohistochemical testing, CDKN2A molecular biology pattern, familial melanoma, and spontaneous multiple primary melanoma in a cohort of Romanian patients.

This analysis highlighted the value of singular p16 immunohistochemical absence as a predictor for aggressive biological behavior and unfavorable prognosis in familial and/or multiple primary melanomas, in comparison with the exclusive loss of p14, indifferent to the histopathological subtype.

Major susceptibility gene in melanoma, with over 60 germinal mutations identified at the moment, CDKN2A alterations mainly imply missense mutations in the p16 transcript.^[10]^ The probability of identifying CDKN2A positivity in families with melanoma aggregation increases proportionally with the number of affected members, the presence of relatives with multiple primary melanomas, pancreatic cancer, or young age at the time of the debut of the malignant melanocytic lesion. However, CDKN2A mutations have been described in patients with multiple primary melanoma, even in the absence of positive familial history (8.3–57% in the US and Greece, respectively, given the fact that the latter is a country with numerous sunny days annually).^[11]^

According to Hayward et al’s study, the most prominent mutation—identified in 56% of tumors—is represented by the hemi- or homozygous deletion of CDKN2A, while its alteration by methylation was detected in a reduced proportion of cases.^[2]^ In our evaluation, CDKN2A homozygous deletion and monosomy were present in the majority of cases, totaling 60.86% of the analyzed melanoma specimens from patients with MPM and/or familial cutaneous melanoma.

In a study conducted by Spagnolo et al, the coexistence of BRAF-positive melanomas in patients with CDKN2A genetic malfunction typically corresponded, in the disease evolutive scheme, to advanced stages, compared to those malignant melanocytic lesions that only harbored the BRAF mutation, detectable from incipient stages.^[12]^ Our investigation identified 9 cases that harbored BRAF gene mutations corroborated with CDKN2A homozygous deletion, CDKN2A monosomic or disomic alterations, among which the majority was associated with distant visceral metastases, therefore sustaining the hypothesis of increased aggressivity and fulminant evolution of melanomas with double mutational load.

As reported by Helgadottir et al, the CDKN2A-mutated (CDKN2A-mut) melanomas showed a less favorable prognosis than melanomas or other cutaneous malignancies that lacked this genetic alteration (CDKN2A-wild type); moreover, no prognostic difference was remarked between CDKN2A-wt and sporadic cases.^[13]^ It has also been proved that CDKN2A deletion and the loss of p16 protein promote tumoral proliferation and the risk of metastasis.^[7]^ Contrastively, the study supervised by Dalmasso et al did not reveal any divergence in global survival and melanoma-specific survival in CDKN2A-mut cases compared with CDKN2A-wt lesions; the explanatory hypothesis of these results was based on the idea of more frequent cutaneous screening, followed by surgical excision of suspicious pigmented lesions in the first category.^[14]^ Concerning the patients comprised in our cohort, 8 out of 10 cases with p16 immunohistochemical absence had metastasized at the time of diagnosis, while the remaining 2 cases with p16 loss did not display distant secondary tumoral foci.

A study by Fątowicz et al found molecular mutations of p14 and p16 proteins in the CDKN2A gene in 95% of melanoma biopsies,^[15]^ a fact that—corroborated with our immunohistochemical discoveries—is congruent with the quasi-perfect relationship with CDKN2A homozygous, heterozygous and monosomic alterations detected via FISH. According to Rizos et al, these positive correlations with molecular tests are derived from the prerequisite existence of p14 nuclear presence that ensures the complete function of p53 in p14^ARF^.^[16]^

A genetic study following the role of genetic pathways associated with CDKN2A in tumor progression and prognosis delineated the significant implication of p16 loss in the progression of melanoma, as opposed to the intermittent role of p14 alterations.^[17]^ In our study, this affirmation was supported by the association between higher Breslow indexes in the case of p16 immunohistochemical absent expression (*P*-value = .0001) than those encountered in melanoma slides with p14 loss (*P*-value = .001). Furthermore, the mitotic rate registered in the p16-loss malignant melanocytic group was 2.64 times higher than the number of mitoses determined in the samples lacking p14 immunohistochemical expression.

Specific screening approaches should be taken into consideration by the dermatology clinician after receiving similar immunohistochemical results, given the fact that they display a particular category of patients, with higher malignancy risks. Therefore, they should be counseled regarding tobacco cessation, visiting a dermatologist for skin examinations every 3 months and ought to be informed concerning the use of sun protective measures.^[18]^

A case report of spitzoid melanoma immunohistochemically testing the p16 tumoral status revealed the absence of p16, connected to a loss of genomic CDKN2A copy.^[19]^ Compared to the limited and sometimes null diagnostic importance of BRAF or NRAS genetic testing in spitzoid melanomas, CDKN2A genetic alterations should be investigated and have been labeled as beneficial in the subsequent therapeutic and secondary prophylaxis management.^[20]^

Dobrowolski et al examined the types of p14 expression in tissue samples from benign melanocytic nevi, primary melanomas and melanoma metastases and detected a notable inverse association between the intensity of p14 immunohistochemical staining and the evolution of cutaneous melanocytic tumoral populations.^[21]^ The interpretation stemmed from the observation of p14 overexpression in almost all of the benign nevi, in comparison with its absence in ¾ of primary melanomas and in all melanoma metastases.^[17]^

Eventually, we must address the limitations of this study, which consist of the small number of cases and the absence of complete histopathological parameters of melanoma-diagnosed relatives of the included patients. However, given the fact that—according to Globocan—approximately 1547 new cases of melanoma are diagnosed annually in Romania,^[22]^ that 0.2% to 8.6% of all melanomas are represented by multiple tumors,^[23]^ that 1% to 8% of all patients diagnosed with melanoma have at least a first-degree relative affected by the same oncological disease,^[24]^ and that our analysis only focused on the Romanian Dobruja region, the correlation between p14, p16, and CDKN2A genetic status is considered statistically significant. More studies that assess the relationship between the immunohistochemical highlight of these protein transcripts and the overall survival under treatment in this populational group should be conducted, in order to determine the efficacy of the available medication and the necessity of developing novel CDKN2A-targeted agents.

## 5. Conclusion

The results of this analysis could represent the starting point for the elaboration of tailored diagnostic and therapeutic algorithms for the Eastern-European population with a suspect or confirmed diagnosis of familial and/or multiple primary melanomas, taking into consideration the unfavorable disease prognosis related to tumoral p16 immunohistochemical loss. The present study also emphasizes the utility of immunohistochemistry techniques as less expensive methods of complementing the provenly accurate testing arsenal that is currently at our disposal for managing these specific patient categories. More studies are necessary to develop specific therapies for CDKN2A-mutated patients, depending on the molecular and immunohistochemical discoveries of the affected proteins.

## Acknowledgments

We would like to acknowledge the aid of Rs. Biol. Sebastian Topliceanu for the technical support regarding FISH testing.

## Author contributions

**Conceptualization:** Luana-Andreea Boşoteanu, Emma Gheorghe, Mădălina Boşoteanu.

**Funding acquisition:** Emma Gheorghe.

**Investigation:** Georgeta Camelia Cozaru, Cristian Ionuț Orășanu, Mădălina Boşoteanu.

**Methodology:** Luana-Andreea Boşoteanu, Emma Gheorghe, Mădălina Boşoteanu.

**Supervision:** Mariana Aşchie, Mariana Deacu, Mădălina Boşoteanu.

**Validation:** Luana-Andreea Boşoteanu, Emma Gheorghe, Mădălina Boşoteanu.

**Writing – original draft:** Luana-Andreea Boşoteanu, Mădălina Boşoteanu.

**Writing – review & editing:** Luana-Andreea Boşoteanu, Mădălina Boşoteanu.
